# Supplementary material for: Space-time wedges
Source: Nanophotonics. 2025 Jan 7;14(23):3939–46. doi: 10.1515/nanoph-2024-0526 (PMC12617698; doi:10.1515/nanoph-2024-0526)
Supplement: Supplementary file 1 — Supplementary Material Details [file j_nanoph-2024-0526_suppl_001.pdf]

Research Article

Amir Bahrami, Klaas De Kinder, Zhiyu Li, and Christophe Caloz*

Supplementary Material for "Space-Time Wedges"

1 Classification of Space-Time Wedges

Figure 1 depicts all the possible types of wedges in a 1+1D configuration.

2 Scattering at Penetrable (Dielectric) Space-Time Wedges

In this section, we derive the scattering coefficients for a wave with arbitrary profile $\psi[\cdot]$ interacting with a space-time wedge.

We start with the general electromagnetic waveforms

$$E_x^{\mathrm{i}} = \psi_{\mathrm{i}}\left[\phi_1^+[z,t]\right], \quad E_x^{\mathrm{r}} = \psi_{\mathrm{r}}\left[\phi_1^-[z,t]\right], \quad E_x^{\mathrm{t}} = \psi_{\mathrm{t}}\left[\phi_1^+[z,t]\right], \tag{1a}$$

$$B_y^{\mathrm{i}} = \frac{n_1}{c}\psi_{\mathrm{i}}\left[\phi_1^+[z,t]\right], \quad B_y^{\mathrm{r}} = -\frac{n_1}{c}\psi_{\mathrm{r}}\left[\phi_1^-[z,t]\right], \quad B_y^{\mathrm{t}} = \frac{n_1}{c}\psi_{\mathrm{t}}\left[\phi_1^+[z,t]\right], \tag{1b}$$

$$D_x^{\mathrm{i}} = \frac{n_1}{c\eta_1}\psi_{\mathrm{i}}\left[\phi_1^+[z,t]\right], \quad D_x^{\mathrm{r}} = \frac{n_1}{c\eta_1}\psi_{\mathrm{r}}\left[\phi_1^-[z,t]\right], \quad D_x^{\mathrm{t}} = \frac{n_1}{c\eta_1}\psi_{\mathrm{t}}\left[\phi_1^+[z,t]\right], \tag{1c}$$

$$H_y^{\mathrm{i}} = \frac{1}{\eta_1}\psi_{\mathrm{i}}\left[\phi_1^+[z,t]\right], \quad H_y^{\mathrm{r}} = -\frac{1}{\eta_1}\psi_{\mathrm{r}}\left[\phi_1^-[z,t]\right], \quad H_y^{\mathrm{t}} = \frac{1}{\eta_1}\psi_{\mathrm{t}}\left[\phi_1^+[z,t]\right], \tag{1d}$$

$$E_x^{\mathrm{w}} = \psi_{\mathrm{f}}\left[\phi_2^+[z,t]\right] + \psi_{\mathrm{b}}\left[\phi_2^-[z,t]\right], \tag{1e}$$

$$B_y^{\mathrm{w}} = \frac{n_2}{c}\psi_{\mathrm{f}}\left[\phi_2^+[z,t]\right] - \frac{n_2}{c}\psi_{\mathrm{b}}\left[\phi_{\mathrm{b}}^-[z,t]\right], \tag{1f}$$

$$D_x^{\mathrm{w}} = \frac{n_2}{c\eta_2}\psi_{\mathrm{f}}\left[\phi_2^+[z,t]\right] + \frac{n_2}{c\eta_2}\psi_{\mathrm{b}}\left[\phi_2^-[z,t]\right] \tag{1g}$$

and

$$H_y^{\mathrm{w}} = \frac{1}{\eta_2}\psi_{\mathrm{f}}\left[\phi_2^+[z,t]\right] - \frac{1}{\eta_2}\psi_{\mathrm{b}}\left[\phi_2^-[z,t]\right], \tag{1h}$$

with

$$\phi_{1,2}^{\pm}[z,t] = \frac{z}{u_{1,2}} \mp t, \tag{1i}$$

where the square bracket symbol, $[\cdot]$, refers to the argument of the wavefunction.

Then, we apply the moving boundary conditions at the first and second interfaces, which are the continuity of $E_x - vB_y$ and $H_y - vD_x$,

$$(E_x^{\mathrm{i}} - v_1 B_y^{\mathrm{i}}) + (E_x^{\mathrm{r}} - v_1 B_y^{\mathrm{r}}) = (E_x^{\mathrm{w}} - v_1 B_y^{\mathrm{w}})|_{z=z_1+v_1 t}, \tag{2a}$$

Amir Bahrami, KU Leuven, Leuven, 3000, Belgium, amir.bahrami@kuleuven.be; 0000-0001-9472-4429
Klaas De Kinder, KU Leuven, Leuven, 3000, Belgium, klaas.dekinder@kuleuven.be; 0009-0003-7646-9016
Zhiyu Li, Xi'an Jiaotong University, Xi'an 710049, China; 0000-0002-0110-0850
***Corresponding author: Christophe Caloz,** KU Leuven, Leuven, 3000, Belgium, christophe.caloz@kuleuven.be; 0000-0003-0502-8435

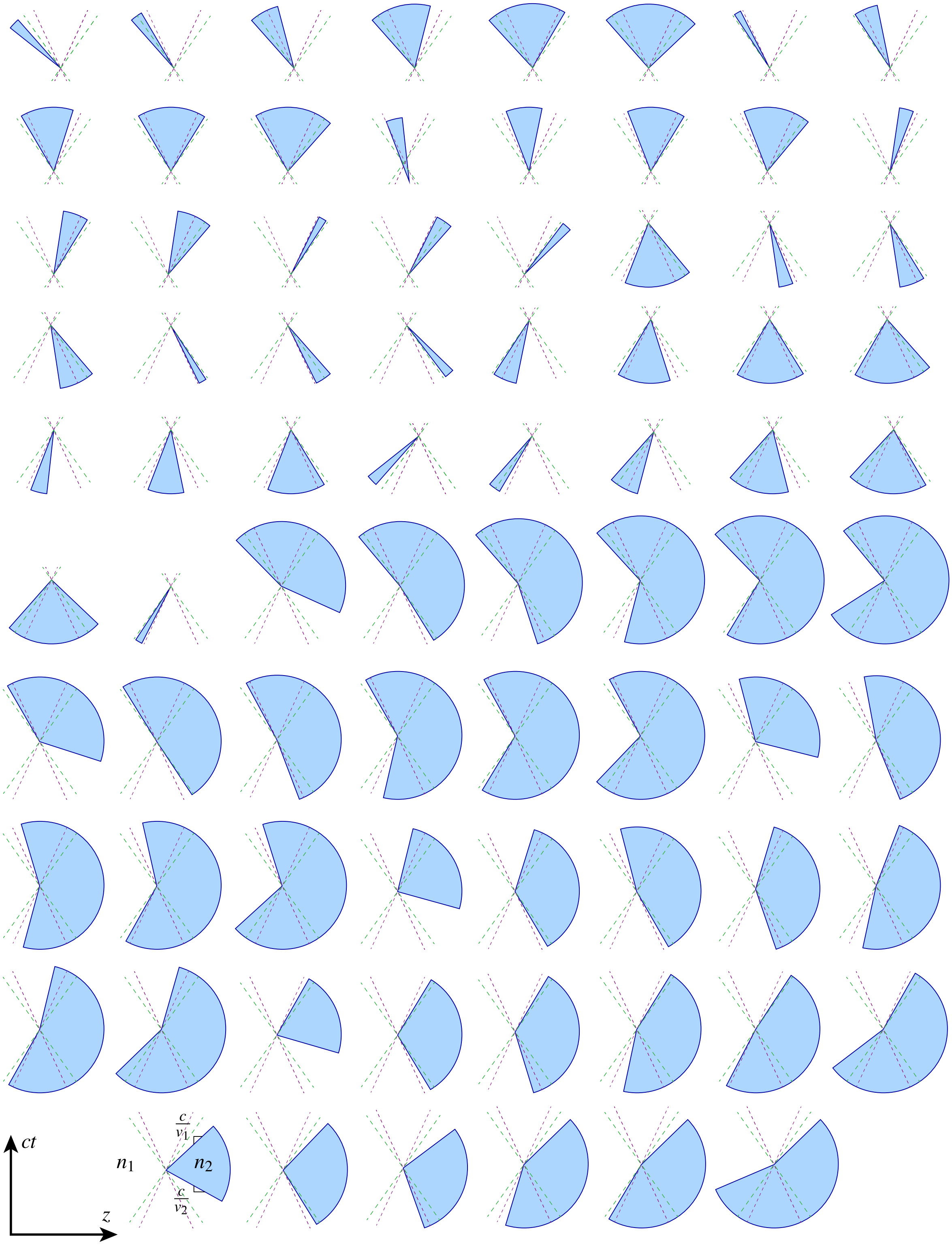

Fig. 1: Classification of space-time wedges.

$$(H_y^{\mathrm{i}} - v_1 D_x^{\mathrm{i}}) + (H_y^{\mathrm{r}} - v_1 D_x^{\mathrm{r}}) = (H_y^{\mathrm{w}} - v_1 D_x^{\mathrm{w}})|_{z=z_1+v_1 t}, \tag{2b}$$

$$E_x^{\mathrm{t}} - v_2 B_y^{\mathrm{t}} = E_x^{\mathrm{w}} - v_2 B_y^{\mathrm{w}}|_{z=z_2+v_2 t} \tag{2c}$$

and

$$H_y^{\mathrm{t}} - v_2 D_x^{\mathrm{t}} = H_y^{\mathrm{w}} - v_2 D_x^{\mathrm{w}}|_{z=z_2+v_2t}. \tag{2d}$$

Substituting Eqs. (1) into Eqs. (2) yields the following equations:

$$M_{11}^{-}\psi_{\mathrm{i}}[\phi_1^{+}] + M_{11}^{+}\psi_{\mathrm{r}}[\phi_1^{-}] = M_{12}^{-}\psi_{\mathrm{f}}[\phi_2^{+}] + M_{12}^{+}\psi_{\mathrm{b}}[\phi_2^{-}]|_{z=z_1+v_1t}, \tag{3a}$$

$$\frac{M_{11}^{-}}{\eta_1}\psi_{\mathrm{i}}[\phi_1^{+}] - \frac{M_{11}^{+}}{\eta_1}\psi_{\mathrm{r}}[\phi_1^{-}] = \frac{M_{12}^{-}}{\eta_2}\psi_{\mathrm{f}}[\phi_2^{+}] - \frac{M_{12}^{+}}{\eta_2}\psi_{\mathrm{b}}[\phi_2^{-}]|_{z=z_1+v_1t}, \tag{3b}$$

$$M_{21}^{-}\psi_{\mathrm{t}}[\phi_1^{+}] = M_{22}^{-}\psi_{\mathrm{f}}[\phi_2^{+}] + M_{21}^{+}\psi_{\mathrm{b}}[\phi_2^{-}]|_{z=z_2+v_2t} \tag{3c}$$

and

$$\frac{M_{21}^{-}}{\eta_1}\psi_{\mathrm{t}}[\phi_1^{+}] = \frac{M_{22}^{-}}{\eta_2}\psi_{\mathrm{f}}[\phi_2^{+}] - \frac{M_{22}^{+}}{\eta_2}\psi_{\mathrm{b}}[\phi_2^{-}]|_{z=z_2+v_2t}. \tag{3d}$$

where

$$M_{ij}^{\pm} = 1 \pm \frac{v_i}{u_j}. \tag{4}$$

We then eliminate ψ_{r} from Eqs. (3a) and (3b) and ψ_{t} from Eqs. (3c) and (3d) to arrive the following two equations

$$T_{12}M_{11}^{-}\psi_{\mathrm{i}}\left[\frac{z_1}{u} - M_{11}^{-}t\right] = M_{12}^{-}\psi_{\mathrm{f}}\left[\frac{z_1}{u_2} - M_{12}^{-}t\right] + RM_{12}^{+}\psi_{\mathrm{b}}\left[\frac{z_1}{u_2} + M_{12}^{+}t\right] \tag{5a}$$

and

$$RM_{22}^{-}\psi_{\mathrm{f}}\left[\frac{z_2}{u_2} - M_{22}^{-}t\right] + M_{22}^{+}\psi_{\mathrm{b}}\left[\frac{z_2}{u_2} + M_{22}^{+}t\right] = 0, \tag{5b}$$

where

$$R = \frac{\eta_2 - \eta_1}{\eta_2 - \eta_1} \tag{6a}$$

and

$$T_{ij} = \frac{2\eta_j}{\eta_i + \eta_j}. \tag{6b}$$

Noticing that the arguments of ψ_{b} in the two relations are different, we perform in Eq. (5b) the change of variable

$$t \to \frac{z_1 - z_2}{u_2 M_{22}^{+}} + \frac{M_{12}^{+}}{M_{22}^{+}}t, \tag{7}$$

which yields, upon substitution, transforms Eq. (5b) into

$$\psi_{\mathrm{b}}\left[\frac{z_1}{u_2} + M_{12}^{+}t\right] = -R\frac{M_{22}^{-}}{M_{22}^{+}}\psi_{\mathrm{f}}\left[\frac{z_2}{u_2} - \frac{z_1 - z_2}{u_2}\frac{M_{22}^{-}}{M_{22}^{+}} - \frac{M_{22}^{-}M_{12}^{+}}{M_{22}^{+}}t\right], \tag{8}$$

which may be substituted into Eq. (5a) to give

$$\psi_{\mathrm{f}}\left[\frac{z_1}{u_2} - M_{12}^{-}t\right] = T_{12}\frac{M_{11}^{-}}{M_{12}^{-}}\psi_{\mathrm{i}}\left[\frac{z_1}{u_1} - M_{11}^{-}t\right] + R^2\frac{M_{12}^{+}M_{22}^{-}}{M_{12}^{-}M_{22}^{+}}\psi_{\mathrm{f}}\left[\frac{z_2}{u_2} - \frac{z_1 - z_2}{u_2}\frac{M_{22}^{-}}{M_{22}^{+}} - \frac{M_{22}^{-}M_{12}^{+}}{M_{22}^{+}}t\right] \tag{9}$$

We can now easily solve this equation by writing it in terms of $x = z_1/u_2 - M_{12}^{-}t$ as

$$\psi_{\mathrm{f}}[x] = X\psi_{\mathrm{i}}[A + Bx] + Y\psi_{\mathrm{f}}[C + Dx], \tag{10a}$$

where

$$X = T_{12}\frac{M_{11}^{-}}{M_{12}^{-}}, \tag{10b}$$

$$A = \frac{z_1}{u_1} - \frac{z_1}{u_2}\frac{M_{11}^{-}}{M_{12}^{-}}, \tag{10c}$$

$$B = \frac{M_{11}^-}{M_{12}^-}, \tag{10d}$$

$$Y = R^2 \frac{M_{12}^+ M_{22}^-}{M_{12}^- M_{22}^+}, \tag{10e}$$

$$C = \frac{z_2}{u_2} - \frac{z_1 - z_2}{u_2} \frac{M_{22}^-}{M_{22}^+} - \frac{M_{12}^+ M_{22}^-}{M_{12}^- M_{22}^+} \frac{z_1}{u_2} \tag{10f}$$

and

$$D = \frac{M_{12}^+ M_{22}^-}{M_{12}^- M_{22}^+}, \tag{10g}$$

whose resolution yields ψ_f,

$$\psi_\mathrm{f}[x] = X \sum_{p=0}^{\infty} Y^p \psi_\mathrm{i} \left[A + BC \sum_{p'=1}^{p} D^{p'-1} + BD^p x \right]. \tag{11}$$

Next, we eliminate ψ_b from Eqs. (3c) and (3d) to arrive at the following equation, expressing ψ_t in terms of ψ_f:

$$\psi_\mathrm{t} \left[\frac{z_2}{u_1} - M_{21}^- t \right] = T_{21} \frac{M_{22}^-}{M_{21}^-} \psi_\mathrm{f} \left[\frac{z_2}{u_2} - M_{22}^- t \right]. \tag{12}$$

We shall now apply a similar procedure [Eqs. (7) to (11)] to determine ψ_b

$$\psi_\mathrm{b}[x] = X' \psi_\mathrm{i}[A' + B'x] + Y' \psi_\mathrm{b}[C' + D'x], \tag{13a}$$

where

$$X' = -RT_{12} \frac{M_{11}^- M_{22}^-}{M_{12}^- M_{22}^+}, \tag{13b}$$

$$A' = \frac{z_1}{u_1} + \frac{z_2}{u_2} \frac{M_{11}^- M_{22}^-}{M_{12}^- M_{22}^+} + \frac{z_2 - z_1}{u_2} \frac{M_{11}^-}{M_{12}^-}, \tag{13c}$$

$$B' = -\frac{M_{11}^- M_{22}^-}{M_{12}^- M_{22}^+}, \tag{13d}$$

$$Y' = R^2 \frac{M_{12}^+ M_{22}^-}{M_{12}^- M_{22}^+}, \tag{13e}$$

$$C' = \frac{z_1}{u_2} - \frac{z_2}{u_2} \frac{M_{12}^+ M_{22}^-}{M_{12}^- M_{22}^+} - \frac{z_2 - z_1}{u_2} \frac{M_{12}^+}{M_{12}^-}, \tag{13f}$$

and

$$D' = \frac{M_{12}^+ M_{22}^-}{M_{12}^- M_{22}^+}, \tag{13g}$$

whose resolution resolution yields ψ_b,

$$\psi_\mathrm{b}[x] = X' \sum_{p=0}^{\infty} {Y'}^p \psi_\mathrm{i} \left[A' + B'C' \sum_{p'=1}^{p} {D'}^{p'-1} + B'{D'}^p x \right]. \tag{14}$$

Consider now Eqs. (3a) and (3b). In these equations, we can express ψ_r in terms of ψ_b (and ψ_i) by eliminating ψ_f, viz.,

$$\psi_\mathrm{r} \left[\frac{z_1}{u_1} + M_{11}^+ t \right] = R \frac{M_{11}^-}{M_{11}^+} \psi_\mathrm{i} \left[\frac{z_1}{u_1} - M_{11}^- t \right] + T_{21} \frac{M_{12}^+}{M_{11}^+} \psi_\mathrm{b} \left[\frac{z_1}{u_2} + M_{12}^+ t \right]. \tag{15}$$

At this point, we have ψ_t and ψ_r in Eqs. (12) and (15) as functions of ψ_f and ψ_b, respectively. The arguments of ψ_t and ψ_r have an awkward form, but they can be forced to the initial traveling-wave forms, as

$$\psi_\text{t}\left[\frac{z}{u_1}-t\right]=T_{21}\frac{M_{22}^-}{M_{21}^-}\psi_\text{f}\left[\frac{z_2}{u_2}-\frac{z_2}{u_1}\frac{M_{22}^-}{M_{21}^-}+\frac{M_{22}^-}{M_{21}^-}\left(\frac{z}{u_1}-t\right)\right] \tag{16a}$$

and

$$\begin{aligned}\psi_\text{r}\left[\frac{z}{u_1}+t\right]=&R\frac{M_{11}^-}{M_{11}^+}\psi_\text{i}\left[\frac{z_1}{u_1}+\frac{z_1}{u_1}\frac{M_{11}^-}{M_{11}^+}-\frac{M_{11}^-}{M_{11}^+}\left(\frac{z}{u_1}+t\right)\right]\\&+T_{21}\frac{M_{12}^+}{M_{11}^+}\psi_\text{b}\left[\frac{z_1}{u_2}-\frac{z_1}{u_1}\frac{M_{12}^+}{M_{11}^+}+\frac{M_{12}^+}{M_{11}^+}\left(\frac{z}{u_1}+t\right)\right].\end{aligned} \tag{16b}$$

These equations can be written in final forms by substituting Eqs. (10a) and (13a) into Eqs. (16), which yields

$$\psi_\text{t}\left[\frac{z}{u_1}-t\right]=T_{21}\frac{M_{22}^-}{M_{21}^-}X\sum_{p=0}^{\infty}Y^p\psi_\text{i}\left[\Delta\phi_p+BD^p\frac{M_{22}^-}{M_{21}^-}\left(\frac{z}{u_1}-t\right)\right] \tag{17a}$$

and

$$\begin{aligned}\psi_\text{r}\left[\frac{z}{u_1}+t\right]=&R\frac{M_{11}^-}{M_{11}^+}\psi_\text{i}\left[\frac{z_1}{u_1}+\frac{z_1}{u_1}\frac{M_{11}^-}{M_{11}^+}-\frac{M_{11}^-}{M_{11}^+}\left(\frac{z}{u_1}+t\right)\right]\\&+T_{21}\frac{M_{12}^+}{M_{11}^+}X'\sum_{p=0}^{\infty}Y'^p\psi_\text{i}\left[\Delta\phi'_p+B'D'^p\frac{M_{12}^+}{M_{11}^+}\left(\frac{z}{u_1}+t\right)\right],\end{aligned} \tag{17b}$$

where

$$\Delta\phi_p=A+BC\sum_{p'=1}^{p}D^{p'-1}+BD^p\left(\frac{z_2}{u_2}-\frac{z_2}{u_1}\frac{M_{22}^-}{M_{21}^-}\right) \tag{17c}$$

and

$$\Delta\phi'_p=A'+B'C'\sum_{p'=1}^{p}D'^{p'-1}+B'D'^p\left(\frac{z_1}{u_2}-\frac{z_1}{u_1}\frac{M_{12}^+}{M_{11}^+}\right). \tag{17d}$$

These equations can be more conveniently written as

$$\psi_\text{t}\left[\frac{z}{u_1}-t\right]=T_{12}T_{21}H\sum_{p=0}^{\infty}R^{2p}D^p\psi_\text{i}\left[\Delta\phi_p+HD^p\left(\frac{z}{u_1}-t\right)\right] \tag{18a}$$

and

$$\psi_\text{r}\left[\frac{z}{u_1}+t\right]=R\frac{M_{11}^-}{M_{11}^+}\psi_\text{i}\left[\phi'_0-\frac{M_{11}^-}{M_{11}^+}\left(\frac{z}{u_1}+t\right)\right]-T_{12}T_{21}H'\sum_{p=0}^{\infty}R^{2p+1}D'^p\psi_\text{i}\left[\Delta\phi'_p+H'D'^p\left(\frac{z}{u_1}+t\right)\right], \tag{18b}$$

where

$$\Delta\phi_p=A+BC\left(\frac{1-D^p}{1-D}\right)+BD^p\left(\frac{z_2}{u_2}-\frac{z_2M_{22}^-}{u_1M_{21}^-}\right), \tag{18c}$$

$$\phi'_0=\frac{z_1}{u_1}\left(1+\frac{M_{11}^-}{M_{11}^+}\right) \tag{18d}$$

and

$$\Delta\phi'_p=A'+B'C'\left(\frac{1-D'^p}{1-D'}\right)+B'D'^p\left(\frac{z_1}{u_2}-\frac{z_1M_{12}^+}{u_1M_{11}^+}\right), \tag{18e}$$

with

$$H=\frac{M_{22}^-M_{11}^-}{M_{21}^-M_{12}^-} \tag{18f}$$

and

$$H'=-\frac{M_{12}^+M_{11}^-M_{22}^-}{M_{11}^+M_{12}^-M_{22}^+}. \tag{18g}$$

3 Scattering at Impenetrable Space-Time Wedges

In this section, we consider an impenetrable (PEC) space-time wedge, excited from its interior by a plane wave with an arbitrary wave profile. We may then write

$$E_x^{\mathrm{i}} = \psi_{\mathrm{i}}[\phi^+], \qquad E_x^{\mathrm{w}} = \psi_{\mathrm{f}}[\phi^+] + \psi_{\mathrm{b}}[\phi^-] \tag{19a}$$

and

$$B_y^{\mathrm{i}} = \frac{n}{c}\psi_{\mathrm{i}}[\phi^+], \qquad B_y^{\mathrm{w}} = \frac{n}{c}\psi_{\mathrm{f}}[\phi^+] - \frac{n}{c}\psi_{\mathrm{b}}[\phi^-]. \tag{19b}$$

where

$$\phi^{\pm} = \frac{z - z_0}{u} \mp (t - t_0) = \frac{z}{u} \mp t - \phi_0^{\pm}. \tag{19c}$$

We apply then the PEC moving boundary conditions at the interfaces, viz.,

$$(E_x^{\mathrm{i}} - v_2 B_y^{\mathrm{i}}) + (E_x^{\mathrm{w}} - v_2 B_y^{\mathrm{w}}) = 0|_{z_2 + v_2 t} \tag{20a}$$

and

$$(E_x^{\mathrm{i}} - v_1 B_y^{\mathrm{i}}) = 0|_{z_1 + v_1 t}, \tag{20b}$$

which, upon substitution, yield

$$\left(\psi_{\mathrm{i}}[\phi^+] - \frac{v_2}{u}\psi[\phi^+]\right) + \left((\psi_{\mathrm{f}}[\phi^+] + \psi_{\mathrm{f}}[\phi^-]) - \frac{v_2}{u}(\psi_{\mathrm{f}}[\phi^+] - \psi_{\mathrm{f}}[\phi^-])\right) = 0|_{z_2 + v_2 t} \tag{21a}$$

and

$$(\psi_{\mathrm{f}}[\phi^+] + \psi_{\mathrm{f}}[\phi^-]) - \frac{v_1}{u}(\psi_{\mathrm{f}}[\phi^+] - \psi_{\mathrm{f}}[\phi^-]) = 0|_{z_1 + v_1 t}, \tag{21b}$$

which simplify to

$$M_{21}^- \psi_{\mathrm{i}}[\phi^+] + M_{21}^- \psi_{\mathrm{f}}[\phi^+] + M_{21}^+ \phi_{\mathrm{b}}[\phi^-] = 0|_{z_2 + v_2 t} \tag{22a}$$

and

$$M_{11}^- \psi_{\mathrm{f}}[\phi^+] + M_{11}^+ \psi_{\mathrm{b}}[\phi^-] = 0|_{z_1 + v_1 t}. \tag{22b}$$

Next, we write Eqs. (22) more explicitly by introducing their arguments as

$$M_{21}^- \psi_{\mathrm{i}}\left[\frac{z_2}{u} - M_{21}^- t - \phi_0^+\right] + M_{21}^- \psi_{\mathrm{f}}\left[\frac{z_2}{u} - M_{21}^- t - \phi_0^+\right] + M_{21}^+ \psi_{\mathrm{b}}\left[\frac{z_2}{u} + M_{21}^+ t - \phi_0^-\right] = 0 \tag{23a}$$

and

$$M_{11}^- \psi_{\mathrm{f}}\left[\frac{z_1}{u} - M_{11}^- t - \phi_0^+\right] + M_{11}^+ \psi_{\mathrm{b}}\left[\frac{z_1}{u} + M_{11}^+ t - \phi_0^-\right] = 0. \tag{23b}$$

We now make the argument of Eq. (23b) identical to that in Eq. (23a) using the following change of variable

$$t \to \frac{z_1 - z_2}{u M_{11}^-} + \frac{M_{21}^-}{M_{11}^-} t, \tag{24}$$

which yields upon substitution in Eq. (23a)

$$\begin{aligned}\psi_{\mathrm{b}}\left[\frac{z_2}{u} + M_{21}^+ t - \phi_0^-\right] = &-\frac{M_{21}^-}{M_{21}^+}\psi_{\mathrm{i}}\left[\frac{z_2}{u} - M_{21}^- t - \phi_0^+\right] \\ &+ \frac{M_{11}^+ M_{21}^-}{M_{11}^- M_{21}^+}\psi_{\mathrm{b}}\left[\frac{z_1}{u} + \frac{M_{11}^+}{u M_{11}^-}(z_1 - z_2) + \frac{M_{21}^- M_{11}^+}{M_{11}^-} t - \phi_0^-\right] = 0.\end{aligned} \tag{25}$$

Eq. (25) can be more compactly written as

$$\psi_{\mathrm{b}}[x] = X\psi_{\mathrm{i}}[A + Bx] + Y\psi_{\mathrm{b}}[C + Dx], \tag{26a}$$

where

$$x = \frac{z_2}{u} + M_{21}^+ t - \phi_0^-, \tag{26b}$$

$$X = -\frac{M_{21}^-}{M_{21}^+}, \tag{26c}$$

$$A = \frac{z_2}{u} + \frac{M_{21}^-}{M_{21}^+}\frac{z_2}{u} - \frac{M_{21}^-}{M_{21}^+}\phi_0^- - \phi_0^+, \tag{26d}$$

$$B = -\frac{M_{21}^-}{M_{21}^+}, \tag{26e}$$

$$Y = \frac{M_{11}^+ M_{21}^-}{M_{11}^- M_{21}^+}, \tag{26f}$$

$$C = \frac{z_1}{u} + \frac{M_{11}^+}{uM_{11}^-}(z_1 - z_2) - \frac{M_{11}^+ M_{21}^-}{M_{11}^- M_{21}^+}\frac{z_2}{u} + \frac{M_{11}^+ M_{21}^-}{M_{11}^- M_{21}^+}\phi_0^- - \phi_0^- \tag{26g}$$

and

$$D = \frac{M_{11}^+ M_{21}^-}{M_{11}^- M_{21}^+}, \tag{26h}$$

whose resolution yields

$$\psi_\text{b}[x] = X\sum_{p=0}^{\infty} Y^p \psi_\text{i}\left[A + BC\sum_{p'=1}^{p} D^{p'-1} + BD^p x\right] \tag{27}$$

or, more explicitly,

$$\psi_\text{b}\left[\frac{z}{u} + t\right] = X\sum_{p=0}^{\infty} Y^p \psi_\text{i}\left[A + BC\sum_{p'=1}^{p} D^{p'-1} + BD^p\left(\frac{z}{u} + t\right)\right]. \tag{28}$$

We apply now the same procedure for ψ_f and find

$$\psi_\text{f}\left[\frac{z}{u} - t\right] = WX\sum_{p=0}^{\infty} Y^p \psi_\text{i}\left[A + BC\sum_{p'=1}^{p} D^{p'-1} + BD^p\left(F + G\left(\frac{z}{u} + t\right)\right)\right], \tag{29a}$$

where

$$F = \frac{z_1}{u} + \frac{M_{11}^+}{M_{11}^-}\frac{z_1}{u} - \frac{M_{11}^+}{M_{11}^-}\phi_0^+ - \phi_0^- \tag{29b}$$

and

$$G = -\frac{M_{11}^+}{M_{11}^-}. \tag{29c}$$

The wave scattered within the wedge is the sum of Eqs. (28) and (29a), which can be expressed as

$$E^\text{w} = \sum_{p=0}^{\infty}(R_1R_2)^{p+1}\psi_\text{i}\left(\Delta\phi_p + (R_1R_2)^{p+1}\left(\frac{z}{u} - t\right)\right) - \sum_{p=0}^{\infty} R_1^p R_2^{p+1}\psi_\text{i}\left(\Delta\phi_p' - R_1^p R_2^{p+1}(\frac{z}{u} + t)\right),$$

where

$$R_1 = \frac{M_{11}^+}{M_{11}^-} \tag{30a}$$

and

$$R_2 = \frac{M_{21}^-}{M_{21}^+} \tag{30b}$$

are the normal scattering coefficients at a PEC interface moving at velocity v_1 and v_2, respectively, and

$$\Delta\phi_p = A + BC\left(\frac{1 - D^p}{1 - D}\right) \tag{30c}$$

and

$$\Delta\phi_p' = A' + B'C'\left(\frac{1 - D'^p}{1 - D'}\right) + BD'^p F \tag{30d}$$

are the accumulated phases for each forward and backward-going wave during the multiple scattering.
